# Supplementary material for: Preparation of Biochar Composite Microspheres and Their Ability for Removal with Oil Agents in Dyed Wastewater
Source: Materials (Basel). 2023 Sep 11;16(18):6155. doi: 10.3390/ma16186155 (PMC10532710; doi:10.3390/ma16186155)
Supplement: Supplementary file 1 [file materials-16-06155-s001.zip › materials-2580152-supplementary.pdf]

Supporting information

S.I. 1. Pore properties of Fe_3O_4@L-ABM$_{500}$

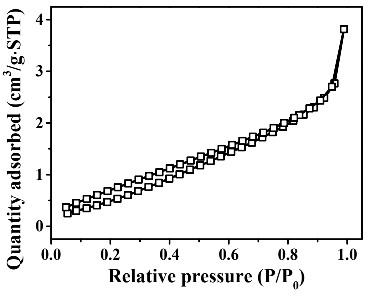

Figure S1 Nitrogen adsorption/desorption isotherm of Fe_3O_4@L-ABM$_{500}$

Table S1 Pore properties of Fe_3O_4@L-ABM$_{500}$.

	S_{BET} (m^2/g)	Pore volume (cm^3/g)	Pore diameter (nm)
Fe_3O_4@L-ABM$_{500}$	3.280 m^2/g	0.003	4.139
